# Effects of Preferred and Non-Preferred Warm-Up Music on Resistance Exercise Performance

**DOI:** 10.3390/jfmk6010003

**Published:** 2020-12-31

**Authors:** Christopher G. Ballmann, Georgia D. Cook, Zachary T. Hester, Thomas J. Kopec, Tyler D. Williams, Rebecca R. Rogers

**Affiliations:** Department of Kinesiology, Samford University, Birmingham, AL 35226, USA; gcook2@samford.edu (G.D.C.); zhester@samford.edu (Z.T.H.); tkopec@samford.edu (T.J.K.); twilli11@samford.edu (T.D.W.); rrogers1@samford.edu (R.R.R.)

**Keywords:** velocity, bench press, motivation, preference

## Abstract

The purpose of this study was to examine the effects of listening to preferred and non-preferred warm-up music on upper-body resistance exercise performance. Resistance-trained males (ages 18–24) participated in two separate bench press trials each with a different warm-up music condition: preferred warm-up music (PREF) or non-preferred warm-up music (NON-PREF). In each trial, participants listened to PREF or NON-PREF music during a standardized bench press warm-up. Following the warm-up, motivation to exercise was measured using a visual analog scale followed by two sets × repetitions to failure (RTF) at 75% of 1-RM separated by 1 min of rest. A linear position transducer was used to measure mean barbell velocity. Rate of perceived exertion (RPE) was obtained after each set. RTF, velocity, RPE, and motivation were analyzed. RTF were significantly higher during the PREF versus NON-PREF trail (*p* = 0.001) while mean barbell velocity remained unchanged (*p* = 0.777). RPE was not significantly different between PREF and NON-PREF trials (*p* = 0.735). Motivation to exercise was significantly higher during the PREF versus NON-PREF trial (*p* < 0.001). Findings show that listening to PREF music during a warm-up improves subsequent RTF performance during bench press exercise. However, barbell velocity was largely unaffected. While perceived exertion was similar between trials, motivation to exercise was markedly increased during the PREF warm-up music trial. These findings suggest that competitors listening to warm-up music before giving maximal effort during resistance exercise could optimize performance by ensuring self-selection of their own preferred music.

## 1. Introduction

Music is widely regarded as an effective ergogenic aid across multiple modes of exercise including endurance, sprint, and resistance exercise [1,2,3]. Various timing strategies of when music is applied have been investigated including pre-task, warm-up, or during exercise which may differentially affect performance benefits [4,5,6]. Regardless, performance improvements from listening to music may be moderated through physiological (i.e., heart rate, neuromuscular fatigue), psychological (i.e., motivation, enjoyment), or psychophysiological (i.e., rate of perceive exertion, arousal) factors [6,7,8]. Recent evidence has further cemented that an important mediator for ergogenic potential of music is musical preference, whereby listening to preferred music improves both performance and psychological factors compared to non-preferred music [1,9,10]. However, whether preferences influence the efficacy of music with different timing strategies is currently less clear.

Anecdotally, warm-up music is frequently used in sport and competition in efforts to improve subsequent performance. While there is a large amount of evidence supporting the use of warm-up music, there are conflicting reports on the degree of efficacy [2,5,11]. Belkir et al. reported improved short-term maximal performance (i.e., 30 s continuous jump tests) following motivational warm-up music both during morning and evening times [11]. Furthermore, Chtourou et al. showed increases in power output during anaerobic cycle sprints in young sprinters following listening to warm-up music [2]. Both physiological and psychological factors have been postulated to be responsible for performance enhancement. Blood catecholamines have been reported to be increased following listening to pre-exercise music which may be responsible for potentiating the sympathetic response to exercise [12]. Supporting this, Arazi et al. revealed that heart rate, systolic blood pressure, and rate pressure product were higher in individuals completing circuit-type resistance exercise after listening to warm-up music [13]. Motivation and rate of perceived exercise (RPE) also appear to play a pivotal role in increasing effort after listening to warm-up music [10,13]. However, others have shown no effect of warm-up music on subsequent performance. For example, Fox et al. reported that despite being self-selected, warm-up music had no influence on anaerobic performance or affective feeling state [5]. Reasons for the disparities between findings are unclear necessitating further characterization of warm-up music and how it influences performance.

A wealth of evidence has shown that individual preference of music largely influences its ergogenic potential [1,3,9,14]. Ballmann et al. reported that listening to preferred music increased repetition volume and barbell velocity during bench press compared to non-preferred music [1]. Supporting this, Nakamura et al. showed increased endurance cycling performance and decreased RPE while listening to preferred music [3]. Lower RPE and increased motivation to exercise with preferred music have also been shown by others during high intensity exercise suggesting that musical preference influences psychological determinants of performance [9]. Recently, Silva et al. reported preferred music genre improves strength, strength-endurance, and lowers RPE during lat-pulldown exercise [14]. However, much less is known about how the timing of preferred music influences performance. To date, almost all investigations on music preference and exercise performance have applied music during the exercise bout leaving the influence of music preference on warm-up music unclear.

Despite the positive findings of preferred music during exercise to enhance performance, listening to music during competition or performance is not viable for many athletes and competitors. Thus, listening to music pre-task or during a warm-up is used in efforts to impose performance benefits. To date, only one study has investigated how warm-up music preference influences exercise performance [10]. Karow et al. showed that preferred warm-up music improved rowing endurance and increased motivation to exercise compared to non-preferred warm-up music [10]. However, it is unknown if these benefits translate to other modes of exercise, namely resistance exercise. Given that strength athletes and powerlifters are among competitors most commonly using warm-up music in competition, knowledge of how musical preference influences subsequent explosive resistance exercise performance is a dire need. The purpose of this study was to examine the effects of listening to preferred and non-preferred warm-up music on motivation to exercise, RPE, and explosive resistance exercise performance.

## 2. Materials and Methods

### 2.1. Participants

Adequate sample size was determined through an *a priori* power analysis using G-power 3.1.9.6 software based off results from a previous investigation on music preference and resistance exercise performance where estimated effect size for barbell velocity improvements were d = 1.6 [1]. Accordingly, the following parameters were used: test = matched pairs *t*-test, d = 1.6, α = 0.05, β = 0.8. Minimum sample size was calculated to be *n* = 6. Resistance trained males (*n* = 10) were recruited to participate in this study. Resistance-trained was defined as accumulating a minimum of 2–3 days of resistance exercise per week [15]. Descriptive characteristics of participants can be seen in Table 1. Prior to participating, screening for safety of exercise was completed using a modified physical activity readiness questionnaire (PARQ). Individuals were excluded if they reported an upper-extremity injury within the last 6 months that disrupted training, cardiovascular disease, musculoskeletal disease, metabolic disease, or other health problems. Prior to each exercise session, participants were asked to refrain from caffeine, nicotine, and alcohol 12 h prior and vigorous upper body exercise 24 h prior [15].

### 2.2. One-Repetition Maximum (1-RM) and Familarization

For the participant’s first visit, one-repetition maximum (1-RM) for bench press was determined and participants were familiarized with lifting with explosive intent. Participants began by warming up with 5 repetitions of 40% of self-reported 1-RM followed by 3 repetitions of 60% of self-reported 1-RM. Each warm-up set was separated by a 3-min rest period. No music was applied for the warm-up for the 1-RM visit. Following the warm-up, the barbell weight was progressively increased by 2.5–20.0 kg for a single attempt until the participant could not complete the concentric phase of the lift [15]. A 3 to 5-min rest period separated each 1-RM attempt. Following this, participants were familiarized with lifting explosively. Participants lifted a standard 20-kg Olympic bar as fast and explosively as possible during the concentric phase for three repetitions for a total of three sets [16]. If needed, form was corrected by researchers.

### 2.3. Music Preference

During the 1-RM and familiarization visit, preferred and non-preferred music was determined using a music preference survey [10]. Participants were instructed to rank order 6 different genres of music (rap/hip hop, rock and roll, country, pop, classical, and alternative) from their most preferred to their least preferred. For the preferred (PREF) music condition, participants were instructed to pick any song with a tempo >120 bpm out of their preferred music genre [10]. For the non-preferred (NON-PREF) music condition, researchers chose a random tempo matched song from the participants lowest ranked least preferred genre. All music was played through headphones using a single electronic device. Volume was standardized to the same level for all participants and all sessions.

### 2.4. Procedures

In a randomized and counterbalanced, crossover study design, participants completed 2 additional visits following the 1-RM trial each with a different condition: (1) preferred warm-up music (PREF), (2) non-preferred warm-up music (NON-PREF). During each visit, participants began by completing a standardized bench press warm-up while listening to the corresponding music condition. The warm-up consisted of 5 reps at 40% 1-RM followed by 3 reps at 60% 1-RM separated by 3 min of rest. Another 1-min rest period was given following the completion of the warm-up and at which time all music was stopped. Motivation to exercise was then measured using a visual analog scale [1,10]. Briefly, participants were instructed to mark on a 100 mm line how motivated they felt to exercise where 0 was “zero motivation” and 100 was “extremely motivated”. Following this, participants then completed 2 sets of bench press × repetitions to failure (RTF) at 75% 1-RM as explosively as possible separated by 2 min of rest. During each set, barbell velocity was measured using al linear position transducer (GymAware; Kinetitech Performance Technology, ACT, Australia). This device has been previously validated for measuring power and velocity during resistance exercise and has shown excellent test-retest reliability in our lab: [ICC] = 0.932 [16,17]. The first 3 repetitions for each set were averaged together to use for barbell velocity analysis. Following each set, rate of perceived exertion (RPE) was determined using a 1–10 scale.

### 2.5. Data Analysis

All data were analyzed using Jamovi software (Version 0.9, Jamovie, Sydney, Australia). A 2 × 2 (condition × set) repeated-measures ANOVA was used to detect set-to-set differences. Tukey post-hoc analysis was used for pairwise comparisons if warranted. Estimates of effect size for main effects were calculated using eta squared (η^2^). Exercise motivation was analyzed using a paired samples *t*-test with estimates of effect size being calculated via Cohen’s d (d). All data are presented as mean ± standard deviation (SD). Significance was set a *p* ≤ 0.05.

## 3. Results

### 3.1. Barbell Velocity and Repetitions to Failure (RTF) Analysis

**Figure 1:**
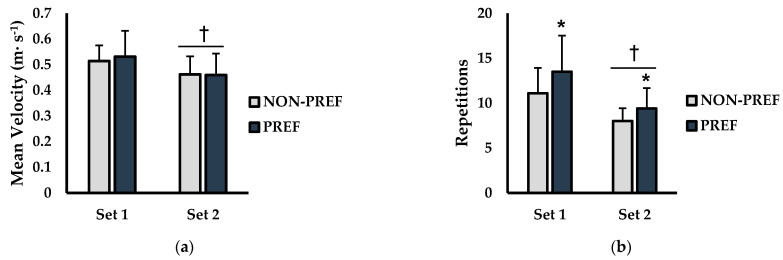
Barbell velocity and repetitions to failure analysis between non-preferred (NON-PREF) and preferred (PREF) warm-up music conditions (**a**) Average mean velocity (m s^−1^) of the first 3 repetitions of each set (**b**) Repetitions completed for each set. Data are presented as mean ± SD. * indicates significantly different from NON-PREF (*p* ≤ 0.05). † indicates significantly different than set 1 (*p* ≤ 0.05).

Mean velocity (m·s^−1^) can be seen in Figure 1a. There was no interaction for condition × set (*p* = 0.499; η^2^ = 0.004) or main effect for condition (*p* = 0.777; η^2^ = 0.001). However, a significant main effect for set was observed (*p* = 0.007; η^2^ = 0.285). Post hoc analysis revealed that mean velocity for set 2 was significantly lower than set 1 for both NON-PREF (NON-PREF-set1 = 0.51 m s^−1^ ± 0.06, NON-PREF-set2 = 0.46 m s^−1^ ± 0.07; *p* = 0.026) and PREF (PREF-set1 = 0.53 m s^−1^ ± 0.10, PREF-set2 = 0.46 m s^−1^ ± 0.08; *p* = 0.017). Repetitions completed until failure (RTF) are displayed in Figure 1b. There was no significant interaction for condition × set (*p* = 0.170; η^2^ = 0.007). There was a significant main effect for condition (*p* = 0.001; η^2^ = 0.051) and set (*p* < 0.001; η^2^ = 0.476). Post hoc analysis for condition showed that repetitions completed were significantly higher during the PREF versus NON-PREF conditions during the first set (NON-PREF-set1 = 11.1 reps ± 2.8, PREF-set1 = 13.5 reps ± 4.0; *p* = 0.002) and second set (NON-PREF-set 2 = 9.4 reps ± 2.2, PREF-set2 = 8.0 reps ± 1.4; *p* = 0.018). Furthermore, repetitions completed were significantly higher for set 1 than set 2 in the NON-PREF (*p* < 0.001) and PREF conditions (*p* < 0.001).

### 3.2. Rate of Percieved Exertion (RPE) and Exercise Motivation Analysis

**Figure 2:**
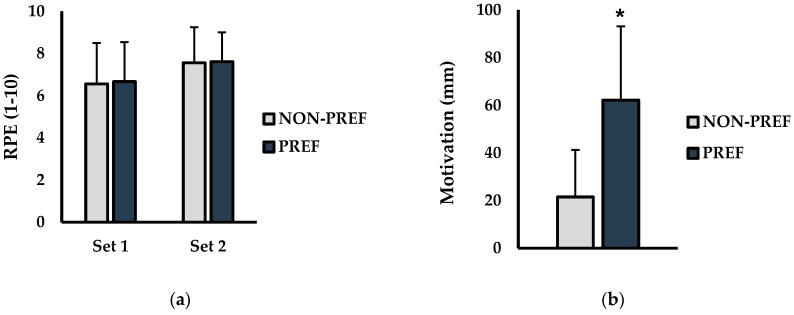
RPE and exercise motivation between non-preferred (NON-PREF) and preferred (PREF) warm-up music conditions (**a**) Set-to-set changes in rate of perceived exertion (RPE; 1–10 scale) (**b**) Motivation (mm) following the warm-up for each condition. * indicates significantly different than NON-PREF (*p* ≤ 0.05).

Rate of perceived exertion (RPE) and exercise motivation are presented in Figure 2. For RPE (1–10 scale) Figure 2a, there was no significant condition × set interaction found between NON-PREF and PREF conditions (*p* = 0.910; η^2^ < 0.001). No significant main effects for condition (*p* = 0.735; η^2^ = 0.001) or set (*p* = 0.127; η^2^ = 0.101) were observed. Additionally, Motivation to exercise (0–100 mm) is shown in Figure 2b which showed that following the warm-up, motivation was significantly higher in the PREF versus NON-PREF condition (NON-PREF = 21.5 mm ± 19.0, PREF = 62.2 mm ± 30.1; *p* = 0.005; d = 1.96).

## 4. Discussion

While listening to warm-up music has been repeatedly shown to improve performance in multiple modes of exercise, conflicting reports still remain [2,5,10]. Music preference has been indicated as an important mediator of ergogenic benefits from listening to music during exercise [1,9]. However, it is less clear if musical preferences influence performance enhancement from listening to warm-up music. Karow et al. recently showed that preferred warm-up music improved endurance rowing performance and increased motivation to exercise compared to non-preferred music [10]. But whether these findings translate to other modes of exercise, namely resistance exercise, is currently unknown. Thus, the purpose of this study was to investigate the effects of preferred and non-preferred warm-up music on upper-body resistance exercise performance. Findings reveal that listening to preferred warm-up music resulted in increased bench press repetition volume and motivation to exercise compared to non-preferred music. However, no differences between conditions were observed for mean barbell velocity or RPE. While causes and mechanisms for current changes remain inconclusive, these results provide novel support for individualizing warm-up music choice to optimize subsequent exercise performance.

In the current investigation, barbell velocity remained unchanged regardless of the warm-up music preference condition. This contrasts with previous investigations comparing listening to preferred versus non-preferred music during resistance exercise. Ballmann et al. reported that barbell velocity and power output were higher when listening to preferred versus non-preferred music during sets of bench [1]. Differences in findings may be due to the removal of the music stimulus before the commencement of explosive exercise in the present study design. Previous evidence has shown that listening to preferred music during exercise may shift attentional focus and cause disassociation to a greater degree than non-preferred music [9,18]. Furthermore, shifting of attentional focus to external factors (i.e., external cues) may improve ballistic exercise and motor performance [19,20]. Taken together, disparities in explosive exercise performance findings may be manifested in a lack of change in external focus to the preferred music since all music was stopped before the explosive bench press exercise. While attentional focus was not directly measured in the current investigation, RPE was not different between warm-up music preference conditions suggesting a lack of alteration in disassociation. This is supported by previous evidence showing that preferred warm-up music resulted in no changes in RPE during rowing exercise [10]. Thus, preferred music may be more beneficial for explosive movements if it is listened to during the performance of the exercise.

Presently, preferred warm-up music resulted in significantly higher bench press repetition volume compared to non-preferred. This reinforces previous findings showing increased repetitions to failure during bench press exercise while listening to preferred versus non-preferred music during exercise [1]. Further supporting these findings, Arazi et al. revealed that listening to warm-up music prior to exercise resulted in faster completion of a resistance exercise circuit [13]. It is plausible that findings may be under control of physiological and/or psychological factors. Yamamoto et al. showed that listening to music prior to exercise resulted in increased blood catecholamines and heart rate [12]. While speculative, preferred warm-up music in the current investigation may have led to an increase in catecholamine release and sympathetic neural activity. Anticipatory increases in catecholamines prior to resistance exercise has been shown to result in optimal muscle force production and resistance exercise performance [21]. Thus, preferred warm-up music may have resulted in a higher anticipatory increase in catecholamines thereby improving performance. However, it should be cautioned that plasma catecholamines were not measured in the current investigation leaving the need for future study on how music preference may influence anticipatory responses to exercise. Psychologically, increases in motivation may have led to the increases in repetition volume. Motivation to exercise has been repeatedly shown to be higher while listening to preferred music compared to non-preferred music [1,9,10]. Importantly, previous reports of increases in motivation while listening to preferred music during exercise accompanied increases in bench press repetition volume [1]. Regarding warm-up music preference, Karow et al. showed that motivation following a warm-up while listening to preferred music increased and accompanied increases in power output during rowing [10]. Increases in motivation may lead to greater effort, as motivational warm-up music has been previously shown to improve maximum effort short-term anaerobic exercise [22]. Taken together, the increased motivation from preferred warm-up music may have led to greater effort and physical exertion thereby improving performance. Interestingly, RPE remained unchanged despite differences in warm-up music preference. Decreased RPE is widely regarded as one of the key performance enhancing mechanisms while listening to music during exercise [3,23]. Previous investigations on warm-up music preference have also reported that RPE remains unchanged during exercise [10]. Likely, this is due to removal of the music stimulus prior to exercise thereby not allowing for a distraction or disassociation effect. Loss of an external focus during the exercise bout may have resulted in little to no shifts in attention. Taken together with previous investigations [10], it appears that the performance enhancing effects of preferred warm-up music is not dependent on changes in disassociation and may be under the control of other physiological and psychological factors. More in-depth study is needed to elucidate distinct mechanisms primarily responsible ergogenic benefits of preferred warm-up music.

Findings of the current investigation reveal important information regarding optimization of performance through the use of warm-up music. However, there were limitations. First, there was not a no music control in the current study design. Thus, findings are only relevant to the comparison of preferred versus non-preferred music and do not give insight into how these fare against no music. However, no music versus warm-up music has already been well described by previous groups with a general consensus of improvements in performance with music [2,10,13]. Also, only a single population (young, trained males) and load (75% 1-RM) was used. As such, findings from the current investigation may not be generalizable to other populations or lighter/heavier loads than that used presently.

## 5. Conclusions

In conclusion, preferred warm-up music increases bench press exercise volume and motivation to exercise compared to non-preferred music. However, barbell velocity and RPE remained unaltered. From a practical standpoint, these novel data support the use of individualized listening of warm-up music prior to competition. Anecdotally, many competitors listen to warm-up music being played over a community speaker for everyone to hear in an arena, on a field, or in locker rooms. While it is possible all competitors prefer the same warm-up music, there is a probable chance of individual variation in music preference. These data suggest that individuals who are subjected to non-preferred warm-up music may have poorer subsequent performance than if they listened to their preferred warm-up music. Thus, athletes and coaches should consider listening to personalized, preferred music in headphones prior to competition in efforts to optimize subsequent performance, particularly with resistance exercise.

## Figures and Tables

**Table 1:** Descriptive Characteristics (*n* = 10).

Characteristic	Mean ± SD
Age (yrs)	21.6 ± 1.7
Height (m)	1.83 ± 0.05
Body mass (kg)	91.56 ± 12.9
Resistance training experience (yrs)	9.0 ± 2.5
Bench Press 1-RM (kg)	105.2 ± 19.1
Relative Strength [1RM (kg)/BM (kg)]	1.2 ± 0.3

## Data Availability

Data are contained and available within this manuscript.
